# Engaging with communities in rural, coastal and low-income areas to understand barriers to palliative care and bereavement support: reflections on a community engagement programme in South-west England

**DOI:** 10.1177/26323524231212514

**Published:** 2023-12-01

**Authors:** Lorraine Hansford, Katrina Wyatt, Siobhan Creanor, Jennie Davies, Gillian Horne, Amanda Lynn, Sheena McCready, Susie Pearce, Anna Peeler, Ann Rhys, Libby Sallnow, Richard Harding

**Affiliations:** Department of Health and Community Sciences, University of Exeter, South Cloisters, St Luke’s Campus, Queen’s Building, Exeter EX1 2LU, UK; Department of Health and Community Sciences, University of Exeter, Exeter, UK; Department of Health and Community Sciences, University of Exeter, Exeter, UK; Patient and Public Involvement Representative; Rowcroft Hospice, Torquay, UK; Torbay Community Development Trust, Torquay, UK; Patient and Public Involvement Representative; School of Nursing and Midwifery, University of Plymouth, Plymouth, UK Torbay and South Devon NHS Foundation Trust, Torquay, UK; Cicely Saunders Institute of Palliative Care, Policy and Rehabilitation, King’s College London, London, UK; Hospiscare, Exeter, UK; St Christopher’s Hospice, London, UK; Marie Curie Palliative Care Research Department, University College London, London, UK; Cicely Saunders Institute of Palliative Care, Policy and Rehabilitation, King’s College London, London, UK

**Keywords:** coastal, community engagement, end-of-life, low-income, palliative care, public health, rural

## Abstract

**Background:**

England’s South-west Peninsula is largely rural, has a high proportion of over 65s, and has areas of rural and coastal deprivation. Rural and low-income populations face inequities at end of life and little is known about the support needs of rural, coastal and low-income communities.

**Objectives:**

To understand how to foster community support for dying and grieving well, a regional, multi-sectoral research partnership developed a community engagement programme to explore experiences of seeking support, issues important to people and the community support they valued. This article shares what people told us about the role that communities can play at end of life, and reflects on learning from our process of engaging communities in conversations about dying.

**Design and methods:**

A programme of varied community engagement which included: the use of the ‘Departure Lounge’ installation and four focus groups with interested individuals in a range of community settings; the co-creation of a ‘Community Conversation’ toolkit to facilitate conversations with individuals with experience of end-of-life care and their carers with Community Builders; a focus group with Community Builders and a storytelling project with three bereaved individuals.

**Results:**

People valued community support at the end of life or in bereavement that offered connection with others, peer support without judgement, responded to their individual needs and helped them to access services. Creative methods of engagement show potential to help researchers and practitioners better understand the needs and priorities of underserved populations. Collaboration with existing community groups was key to engagement, and contextual factors influenced levels of engagement.

**Conclusion:**

Local community organizations are well placed to support people at end of life. This work highlighted the potential for partnership with palliative care and bereavement organizations, who could offer opportunities to develop people’s knowledge and skills, and together generate sustainable solutions to meet local need.

## Introduction

The South-west Peninsula (SWP) region of England (Devon, Cornwall and Somerset) is a largely rural region with an extensive coastline. Data analysis completed by the National Institute for Health Research (NIHR) Clinical Research Network (CRN) SWP Business Intelligence Unit^1–3^ revealed that compared to other CRN areas in England, in 2018 the SWP had the highest percentage of population aged 65 and above (24.2%) and the highest national average index of multiple deprivation score for rural villages and dispersed populations (17.7%). The Chief Medical Officer’s (CMO) 2021 Annual Report^4^ identified coastal areas as having the worst health outcomes in England, with low life expectancy and a higher burden of disease across a range of conditions, including, for example, Coronary Heart Disease and Chronic Obstructive Pulmonary Disease. The report describes a ‘coastal excess’ of disease which remains even when age structure and deprivation levels are accounted for. It concludes that coastal communities have been long overlooked with limited research on their health and well-being. Challenges in accessing healthcare for those living in rural and remote areas are exacerbated at the end of life.^5^ Both rural populations and those with lower socio-economic status experience inequalities not only in accessing healthcare^6^ but more generally at end of life (e.g. social isolation, financial impact).^7,8^ Moreover, populations experiencing health inequalities are underrepresented in health and medical research, thereby limiting the voice of those with the highest burden of illness in contributing to research which seeks to understand these differences.^9^

Interest in public health approaches to palliative care has grown, partially because community involvement in caring for the dying and bereaved is one potentially sustainable response to a growing need for care and support at the end of life,^10^ particularly for those most impacted by structural disadvantages in accessing healthcare.^11^ Public health or health-promoting approaches to palliative care are heterogeneous both in terms of the terminology used and the range of practices described.^10^ Sallnow and Paul^12^ define community engagement in end-of-life care as

a process which enables communities and services to work together to understand, build capacity and address issues to improve their experience of end-of-life and bereavement and their related well being. It exists on a spectrum of engagement that extends from informing through to empowering, depending on a range of factors such as the degree of participation from the local community and the intention of the work.

Kellehear’s model of ‘compassionate communities’ has become a globally known public health approach to palliative care,^13^ with some examples of local implementation within the United Kingdom, such as Compassionate Inverclyde.^14^ Within the SWP, St Luke’s Hospice, with Plymouth City Council, led an initiative to become the first ‘Compassionate City’ in England.^15^ Other examples of innovative practice include Torbay Community Development Trust’s (TCDT) work which uses an Asset-Based Community Development approach to support healthy ageing within communities. This was highlighted as a case study in the CMO’s Coastal Health report.^4^ However, as Grindrod notes, frameworks that support a shift in policy and practice towards a public health approach that intentionally targets underserved populations are still in their infancy.^11^ Little is known about the needs of rural, coastal and low-income communities within a public health approach to palliative care.

In January 2022 a new South-west Peninsula Palliative Care Research Partnership (SWPPCRP) was formed, with the aim of establishing a multi-sectoral collaboration with the capacity to identify and respond to palliative care research needs in the region. The partnership had a particular interest in how community support for dying and grieving well might best be fostered. Funded for 15 months by the NIHR, the partnership brought together academics and practitioners from four universities, seven hospices, organizations and individual community members from the voluntary and community sector with an interest in health and social care (see Appendix 1). To better understand the support needs of communities in underserved rural, coastal and low-income areas at the end of life, SWPPCRP members carried out a scoping review of existing evidence reporting public health palliative care interventions that enable communities to support people who are dying and their carers.^16^ We also developed a programme of community engagement to explore:

- What issues are important to people in this area when they think about death and dying?
- What information/support are people seeking?
- How accessible, useful and relevant do people find the resources and services available locally?
- What are the issues and contextual factors particularly related to rural, coastal and low-income communities that affect people’s experiences of end of life?
- How should these findings inform research priorities locally?

A table of themes from the findings is shown in Appendix 2. Data related to experiences of accessing healthcare and support at end of life, as well as the development of the partnership and future plans, are reported elsewhere.^17^ Given the relatively small scale and ‘snapshot’ nature of our engagement programme, a formal evaluation was not included. However, given that there is little literature reporting informal community engagement, particularly in rural and coastal areas, we felt that it may be helpful to share our learning from this process. The purpose of this article is to share what people told us about the role that communities can play at end of life, and to reflect on learning from our process of engaging communities in conversations about dying.

Whilst there is often a distinction in the literature, and practice, between ‘end-of-life care’ (as treatment and support for people in the last few months, weeks or days of their life) and ‘palliative care’ (as an earlier intervention for those diagnosed with a life-limiting illness), much of the data reported in this article refer to broader conversations about living with life-limiting diagnoses, dying and bereavement, in which participants were unlikely to make such distinctions. We therefore use the terms ‘end-of-life care’ or ‘palliative care’ when the data clearly denotes a specific service, and terms such as ‘support at end of life’ to reflect the broader context in which participants discussed their experiences.

## Methodology: Our community engagement strategy

We carried out a range of activities over a 10-month period aiming to engage individuals not currently in contact with, or aware of, palliative care services, as well as those with lived experience of accessing support at the end of life or in bereavement. Methods were chosen for pragmatic reasons based on timescale, staffing capacity (one researcher for 1 day/week), available resources [such as the Departure Lounge kit (9)] and that they could utilize existing relationships between the research group and community organizations and generate new links facilitated by partnership members.

Phase I of the programme (January–August 2022) focused on informal and exploratory engagement and observation activities. In phase II (July–October 2022) the issues raised were explored in more depth through focus groups and storytelling.

### Phase I

#### The Departure Lounge

The ‘Departure Lounge’ is a pop-up installation produced by the Academy of Medical Science (9), designed to promote conversations about death and dying. Using the metaphor of an airport Departure Lounge, it includes visual resources (e.g. suitcase labels, postcards, printed towels) to disseminate information and provoke discussion. The researcher (LH) used the display in a range of venues around the region, purposively including rural, urban, coastal and inland locations. Settings were suggested by members of the partnership or their contacts. Potential hosts were contacted by LH to explore suitability and interest, with visits arranged to those who responded positively. Nine visits were made in total; four at specific events – a bereavement open day in a cathedral, a Dying Matters Awareness Week event in a health centre, a cross-sector neighbourhood family fun day on a school campus and a Public and Patient Involvement (PPI) group meeting (see below). At others the display was available at the venue (e.g. in community cafes, charity shops) whilst usual activities took place. After each session LH made (anonymized) field notes about the level and type of engagement with the display, and the topics discussed.

#### Community Conversations

‘Community Conversations’ aimed to draw reflections from participants with lived experience of end-of-life care and their carers. As it was not feasible to carry out this workstream across the peninsula, Torbay was chosen as an area in which TCDT, a partnership member organization, had strong links within the community, and as a local authority ranked as the 48th most deprived district in England (out of 317).^3^ TCDT commissioned a local creative producing house (Filament) with whom they had previously collaborated, to create an engagement tool that could be used in people’s homes to facilitate conversations. Filament curated a vintage suitcase containing object-based prompts and simple creative activities (see Appendix 2). The activities incorporated seven questions considering: identity and how I see myself; my experience of end-of-life care; the circle of support around me; how where I live has informed my experience; the support I need (physical, emotional, spiritual, financial, practical); what I wish for others experiencing end-of-life care and what are the essential elements for connected support for good end-of-life care within communities. Participants could choose not to answer any question, or to answer verbally without engaging with the activities.

TCDT employs a team of Community Builders (CBs) whose role is to become embedded in neighbourhoods to promote connection, activity and collective support. CBs were invited to a training session to trial use of the suitcase. The initial design was tested with three participants and refined in response to feedback. Six CBs then carried out Community Conversations, using their existing contacts to identify suitable potential participants, approach them to explain the project and invite them to take part. Twenty-six individuals participated, and informed consent was obtained. Age, gender and ethnicity demographics are shown in Appendix 3. Most conversations lasted between 90 and 120 min, though one ran over three meetings taking a total of 5 h. Responses from Conversations were captured (in text) by the CBs during the session, in discussion with the participant. The notes were then pseudonymized and entered onto a database. LH then made summary notes for each ‘case’ under five headings aligned with the research questions: context, key issues, positives, what could be better and essential elements for supporting end-of-life care within communities. After completing the Conversations, the CBs were invited to a focus group to elicit their views on the key issues discussed, and their reflections on facilitating the conversations. All six agreed to participate.

### Phase II: July–October 2022

#### Focus groups

A convenience sampling approach was used to recruit participants to the four focus groups. Community organizations with whom the partnership had existing links were approached and asked if they would be interested in hosting a focus group. Those who responded positively were followed up, with care taken to ensure that the locations varied across different geographical and socio-economic areas and purposefully included one carer organization. The host settings included:

- a community group supporting people living on a low income in a small coastal town
- a care organization in a small market town serving a rural inland area
- a carers’ organization in a coastal city
- a community café in a low-income neighbourhood in a coastal town

The host agencies advertised the ‘Dying and Bereavement Focus Groups’, inviting those with experience of, or views about; accessing care or support with a life-limiting illness, caring for someone at end of life, getting support in rural, coastal or low-income areas, money, housing or other worries at end of life, or how communities can offer support. Interested individuals were invited to contact either the organization or the researcher and were then sent an information sheet. A total of 22 participants attended. Informed consent was obtained at the beginning of the session and age, gender and ethnicity demographics are shown in Appendix 4. The length of focus groups ranged from 66 to 116 min, and discussions were audio recorded and transcribed.

#### Storytelling

Filaments were commissioned to work with three Community Conversation participants in more depth to create narrative stories about their experiences of living with or caring for someone with a life-limiting illness. CBs approached participants they felt might be interested and whose stories would illustrate a range of different experiences of care at end of life. Three participants agreed and their stories have been captured through one-to-one meetings with the storyteller. The creative production is ongoing to date.

#### PPI group

During the formation of the SWPPCRP, member organizations publicized the partnership, and the opportunity to participate in a PPI group, with members of the public with whom they were in contact. The aim of this group was to include the voices of people with experience of accessing palliative care services in the Steering Group’s discussions, planning and decision-making. Eight individuals expressed an interest, and all were invited to an initial meeting to find out about the partnership and discuss the potential role of the group. Six people attended and subsequently regularly participated (five from different hospices and one from a community organization). Each member had experienced caring for a family member at end of life. Members were paid £25 per hour in recognition of the time they spent engaged in partnership-related activities. PPI members were invited to all (online) partnership Steering Group meetings and chose to hold their own separate meetings in person. PPI members participated in planning the engagement events, helped facilitate the workshop described below, and ran a training session to feed back to the partnership their views on the key issues for those accessing support at the end of life.

#### Sense-checking workshop

In October 2022 the partnership ran a ‘sense-checking’ workshop, presenting findings from the engagement activities and evidence from the scoping review. Steering Group members were invited together with other organizations who had expressed an interest in the partnership’s work. Twenty-seven individuals participated: 11 staff from 5 hospices, 5 PPI members, 7 academics from 4 universities, 2 individuals representing community organizations and 2 from end-of-life charities. Attendees discussed the findings and priorities for future research and service development.

#### Analysis

Field notes from the Departure Lounge sessions, notes from the Community Conversations, PPI session and sense-making workshop, and focus group transcripts, were coded using Lumivero’s NVivo 11 Software. Analyses combined both a deductive approach informed by the research questions and focus group topic guides (see Appendix 6), and an inductive approach deriving codes from the data. As there was significant concurrence between the codes related to community support derived from data collected from community members (i.e. focus group transcripts and Community Conversation notes) and those derived from reflective field notes, an overall framework of themes and subthemes was created (Appendix 2). Insights related to the engagement process are therefore reported alongside findings from the data in the following sections.

### What did we learn about the role of communities in supporting dying and grieving well?

Although people had differing experiences and levels of support, there were some commonalities in the aspects people valued, and these elements were echoed in reflections from our engagement activities. Relevant observations about the process are therefore woven together with findings from the data reported here. The subsequent section looks specifically at the ways in which the creative tools we used helped facilitate discussion and explores facilitators and barriers to engagement.

#### Connection

Making connections with others reduced feelings of isolation, this was particularly the case for family carers or the bereaved. Within the focus groups and Community Conversations, some people described feelings of relief or comfort from talking to others in a similar situation, mentioning carers groups or community groups; others valued taking part in activities in their local neighbourhood. The benefits of social connection, and in particular the value of social situations in which it was ‘acceptable’ to discuss illness, dying and bereavement, were also evident in the way some people reacted to their participation in the engagement activities. Several CBs felt that the Conversations provided a positive experience for participants:

I think sometimes it’s quite therapeutic for the person because actually it’s a chance. Like one of my ladies, she said ‘Do you know what? That’s the first time I’ve actually spoken about it for a while’, so actually it was quite good for her.

[CB Focus Group]

The research process itself appeared to create spaces for social connection. Within all of the focus groups, participants commented that they found the focus group a helpful opportunity to talk about dying and bereavement that they did not feel was available elsewhere:

Actually it’s helped me, so I can’t speak for everybody around the table, but I’ll go away a happier person, because I don’t talk to anybody outside the family, I just don’t have time.

I’ve been walking on eggshells because I just didn’t know what to do, what to say [. . .] But it’s like I needed someone to talk to because I didn’t want to speak to [name] because I knew what she was going through. And I just didn’t know where else. . .

This is my first time here, and I feel like a whole weight has been lifted off my shoulders already.

[Participants from 3 Focus Groups]

#### Peer support and feeling accepted

Feeling able to express emotions without feeling judged was also identified as an important aspect of community support. For example, some carers described the relief they felt after sharing their frustrations and feelings of guilt with others in a similar situation:

R1 I feel like it’s not just me. That’s the thing, it’s not just me. ‘Oh my God, she understands!’
R2 I’m not the only one!
R1 Exactly
R2 I’m not an evil person for saying something.
[Focus Group 4]

In both focus groups held in low-income neighbourhoods, participants described community groups run by staff or volunteers from within the community as places where they did not feel judged and so felt more comfortable seeking support than they did from larger organizations or professional support services:

R1 This is a safe space to talk about their experiences and not feel judged. That’s how I feel.
R2 Whatever you say sounds normal, if that makes sense.
R1 People in here do not have to pretend to be happy or pretend to be something they are not, or do not have to do certain things to fit in, or have money, and so all the barriers to normal places are left at the door.
[Focus Group 3]

One community group discussed their feelings about a local care charity, which they perceived to be geared towards local residents who were able to donate to the organization. Although the charity did have provision for people unable to pay, the group expressed the view that many people on a low-income feel uncomfortable using the service and were unlikely to ask them for help as ‘you don’t feel like your face fits’. The group compared this to their own approach, which they describe as mutual peer support, and upskilling within the community. As well as building resilience, some of the group felt that the support they received from each other was of a different quality (compared to support from a professional) because of the nature of the voluntary relationship, which felt more equal.

Similarly, some other people also talked about support services or charities which had not felt helpful for them because they did not share a common background or experience:

But I had [bereavement charity] myself and I had a very old lady turn up and there was just no connection. I couldn’t connect with her, and she couldn’t connect with the way I’d lost my partner. So that didn’t help me, and I had to travel to [city] to find a group of people that, when I walked in the room, I felt like, you know what I’m going through.

[Focus Group 2]

#### Responding to individual needs

People found support more helpful when the organization or group responded to their needs or concerns, rather than following a service-led process. For example, one person valued the help they received from a local carer’s organization because they offered ‘support that you can opt in or out of’, time to listen without being intrusive and were ‘not fixing but listening’.

Some community groups in low-income areas felt that a flexible offer that responds to the individual, often in a face-to-face setting, is key in terms of making support accessible to people who may find it difficult to ask for help or who may be put off by the systems used by larger organizations. One volunteer compared this to her own group’s daily drop-in, in which anyone can call in for a free coffee and a chat, and volunteers respond to any needs that people discuss:

So, then it’s making an appointment, which you might not feel like that in seven to ten working days, filling out a form, and fitting the criteria. We don’t have criteria for who walks in here, you don’t have to tick a box to fit in with what we do.

[Focus Group 3]

In another focus group, a participant described how they felt a similar informal approach made it easier to access help:

Yes, and [name] and [name] who was here earlier, they always help you with form filling. I struggle with forms [. . .] They say ‘Oh, do it online’. I can’t do it online because I’ve only got an iPad and half the time they don’t understand that you can’t do them on that, but it’s so good to talk. You come in here, cup of tea, cup of coffee, juice or whatever, and you’re not pressurised into saying anything.

[Focus Group 1]

In one focus group in a rural area, a community worker stressed the importance of understanding why someone might not reach out for help. She reflected that, from her experience, some people who have not received positive support from services in the past can particularly find it difficult to seek help in bereavement. The group discussed the problematic nature of ‘one size fits all’ approach to community support, and the need for multiple ways for people to connect:

So it’s not like a delivery service, it’s not like an advert through the door. Maybe lots of different – it’s having lots of different layers, isn’t it, to one’s community, to one’s networks. So if you bounce off one, you can maybe be caught by another so that the system is much more flexible.

[Focus Group 2]

#### Independence

Alongside the importance of community groups creating connections for those who might not be aware of, or feel comfortable approaching, services or organizations offering support, people also described voluntary sector organizations playing an advocacy role when they were unable to access the services they needed. A number of people described their experiences of having to ‘fight’ to obtain appropriate health or care support, or welfare benefits, when a family member was living with a life-limiting illness. Some volunteers or staff in the voluntary and community sector felt they were able to advocate in such situations, and that their independence from statutory provision meant that they were able to challenge services that were not meeting an individual’s needs.

Some people also discussed the value of being able to talk about their feelings and experiences with people outside of their immediate family or networks, often because they wanted to protect others’ feelings and sometimes because there were tensions or different views within the family. Often, people did not want to feel that they were ‘burdening’ other people, for example, friends or work colleagues, by turning to them for emotional support.

#### Emotional impact of providing informal support

Volunteer and paid community workers who were part of the focus groups acknowledged that they sometimes find it emotionally demanding to support people at the end of life or in bereavement. Sometimes this was because they were concerned that they would not be able to provide the right support or that there may not be sufficient support available:

I think that’s one of the things that being part of these communities, that we sometimes are trying so hard to keep everybody else together that we’re falling apart in the background, and always wondering whether we’re doing enough or the right thing.

[Focus Group 1]

Community workers recognized that providing support can be both rewarding and challenging. A community worker described being motivated by her own experience to run a grief café, but also finding it hard:

It’s an incredible thing to be part of. I find it very difficult. I’ve struggled the last few weeks because of my own grief, and I find myself thinking why am I doing this twice a year? But then I come back to, like now, and I realise how important it is to be able to do that. And it turns people’s grief around.

[Focus Group 2]

This ambivalence was also reflected in the CBs’ experiences. Several described it as an ‘honour’ to talk with people about death and reflected that it had been a learning process that added a positive dimension to their practice:

I think going through that journey with them, it’s kind of helped me as well with, about death. I said to [name], ‘Have you ever seen someone go?’ She said ‘Oh yes, loads of people’. [Laughter] [. . .] It makes you realise that yes, it is just the circle of life [. . .] So it’s made me think about how I see it all and what is missing.

[CB Focus Group]

CBs acknowledged that although they felt that the Conversations had been a positive experience, it had sometimes been upsetting, and could be emotionally exhausting:

I realised that because I went through a lot last year myself, I actually got to the point in the middle of it where I nearly burst into tears myself and I had to really hold myself together, and it came out of nowhere. It was just one thing that she said that I really related to [. . .] So I think I have struggled a little bit with it.[. . .] But it was really good.

[CB Focus Group]

CB’s reflected that being involved in the partnership project gave them the opportunity to develop skills, experience and a greater confidence to engage in conversations about dying and bereavement. However, the timeframe imposed by the research was unhelpful as it contributed to the intensity felt by some CB’s carrying out up to eight conversations within a short period.

The group also reflected that it been valuable for them to work together on the project, learning together and also debriefing after carrying out the Conversations. They all felt that it would be important for any practitioners or volunteers doing similar work in the community to have supervision or peer support in place to ensure that it was sustainable in terms of their well-being:

They’d have to have somebody to check in on them afterwards as well; if you had a volunteer coordinator, you need somebody at the end to just say ‘How did that go? Are you alright? How are you feeling?’ Because I feel like we’ve all got each other [. . .], so I think as long as you can reflect back with somebody at the end of the day, I think that would be a really good thing.

[CB Focus Group]

### What did we learn about engaging underserved populations in conversations about end of life?

#### Using creative tools

##### The Community Conversations suitcase

TCDT initially envisioned that the Conversations would be carried out by both CBs and any existing volunteers who wished to take part in the training, which was offered to 15 CBs and 4 volunteers. Six CBs chose to take part, and no volunteers. Reasons given for declining included time capacity, feeling uncomfortable with the subject, feeling that the people they were working with would not wish to take part or not wanting to recruit participants themselves.

Generally, CBs found it quite difficult to recruit participants. Twenty-six conversations took place out of the original target of 40 which CBs had judged to be feasible, and these were with participants with whom the CBs had an existing relationship. Because of their role within neighbourhoods, CB’s were aware of individuals circumstances, whether the activity might be appropriate for them, and often made a judgement about whether it would be sensitive to invite them to take part (taking into consideration, e.g. the length of time after a bereavement). CBs reflected that difficulties with recruitment were partly due to the short (3 months) timescale and that they were aware of other potential participants whom it would have been appropriate to approach in later months.

Feeding back on the design of the Conversation tool in their focus group discussion, the CBs felt that the ‘suitcases’ were useful for facilitating discussion about dying and bereavement, which they felt could otherwise be a difficult topic to broach. They found that the creative activities acted as prompts for reflective conversations as they ‘put thoughts in people’s heads and get them to think about certain issues’. They observed that some participants liked having something to hold or do with their hands (such as knot tying), during difficult conversations. Having playful objects such as Lego could ‘change the mood’ within conversations, and sometimes introduced humour, which helped build rapport:

When I started getting out the objects, I was like ‘Bear with me, this will make sense in a minute. [Laughter] We’re not building anything here’. And they were sort of like ‘Oh, what’s going on here?’ kind of thing, it changed the mood again.

[CB Focus Group]

CB’s felt that the suitcases needed to be used by skilled facilitators, who could ‘read’ the reaction of the participant to different activities and tailor them so that the individual felt comfortable:

All the bits in there are quite useful, but when you’re somebody that’s used to speaking with different people [. . .] you can actually tailor it a bit and you can get that feeling [. . .] that’s not helping this particular interview, so you try and leave most of it behind [. . .] not have it as a rigid ‘We have to do every single bit on this’.

[CB Focus Group]

One CB felt that the activities would be useful if she was carrying out the conversations with people she did not know, but that they could feel unnecessary or even unhelpful with participants with whom she already had a relationship:

Most of the people I did, I knew them and they would have been happy to just have a conversation with me, [. . .] they didn’t need any of that. In fact, some of them would say ‘Behave, what’s all this?’ And sometimes I would say ‘Okay, there might not be an object there, but have a look around your room; is there something that you. . .?’ [. . .] But on the whole I felt it was a distraction.

[CB Focus Group]

Most CBs particularly valued the final activity, in which participants planted sunflowers and chose five ‘essential elements’ for good end-of-life care, because it provided an opportunity to summarize and check their understanding of the conversation, and also left the participant with a gift which was seen as a positive symbol of life. They felt that many participants were keen to influence change by articulating what had worked for them and what could have been better. For some participants the sunflower also provided a talking point for continuing contact with the CB:

The best thing, like you say at the end, the sunflower, they’ve all come back to me and gone ‘Oh, my sunflower’s growing’, [laughter] which is lovely. So I think you’re almost giving something – which was the idea, obviously – but the fact that they were left with something that was growing, that was alive, I thought that was a nice touch and I think people appreciated it.

[CB Focus Group]

Although the ‘suitcase’ receptacle was chosen with the intention that the symbolic opening and closing of the case would help to safely bracket conversations, this was not found to be relevant, as the CBs felt that it was their relational skills that enabled them to hold the conversation with care. CBs reflected that they were aware that they were asking people to discuss an emotional topic, sometimes surfacing feelings of grief and were careful to end each session sensitively:

I asked as well towards the end of it all, like when they were finishing all the case stuff, just asking them ‘So what are your plans this evening? Have you got anything on?’ just trying that but also make sure have they got people around them and doing something, they’re not just sat there on their own thinking about it all.

[CB Focus Group]

CBs felt it was important to follow-up each Conversation with a phone call within a few days to check in with the participant.

Overall CB’s described using the suitcase as a positive experience, and felt that the discussions that had emerged were valuable for the research. Some felt that being given a ‘mandate’ to have conversations about dying and bereavement gave them both permission and encouragement to raise what can be seen as a sensitive topic, as well as a toolkit to do so.

##### The ‘Departure Lounge’ installation

The display is large and colourful and was often successful in attracting attention in public spaces, though levels of engagement varied in different environments (see below). The use of the ‘Departure Lounge’ theme meant that people were not immediately aware of the display’s subject matter when they approached it, which prompted questions and provided openings for discussion. The Departure Lounge theme provoked mixed reactions; some people found it amusing or interesting, and a small number found it distasteful; this was echoed in our PPI group, some of whom liked it, whilst others felt that the phrasing might feel harsh or insensitive, particularly for people recently bereaved.

Overall, the resource was most useful as a starting point for engagement. Occasionally, people took leaflets and asked questions, so it sometimes played an informative role. No one chose to participate in the interactive elements, for example, to complete a postcard about their experiences or wishes, although some took them away. The tool could be used to ascertain needs in different settings (e.g. people requested advice about how to support bereaved children, or how to deal with legal matters such as wills or power of attorney on a low budget), which could be followed up with tailored provision. Several representatives from different organizations (e.g. a hospital chaplain team, a community association) commented that they felt such a resource would be helpful to provoke conversations about death and dying in their own settings.

### Facilitators and barriers to engagement

#### Place and context

Engagement with the Departure Lounge was higher in informal settings that people were familiar with, and where informal social interactions are the norm, such as the community cafes. There was less interaction with the installation in venues such as charity shops or the health centre, where people had come into the space for a different and specific purpose. Considering who normally comes to the space and their intention is therefore important in choosing settings to engage people.

Given the unusual nature of a public engagement activity about dying, some people asked whether there was a religious, or commercial, motive behind the installation (e.g. selling funeral products or promoting a religion). This could potentially be reinforced by its location, for example, in a church hall or shopping centre. So similarly, it is important to consider how the assumptions that people might make about the purpose of the display can be influenced by the setting. The connotations that can be attached to a place or organization were also discussed in one focus group, when a community worker reflected on how perceptions of a grief café shifted when the organizers changed:

Part of us taking it over has helped actually, because when it was church-based, that did put some people off. Because then they expect it to be a religious aspect and we’ve been able to take that away, which is good.

[Focus Group 2]

#### Relationships

In all of our engagement activities, it was clear that they worked most effectively when the researcher was collaborating with individuals and community groups that had existing, long-term relationships within local neighbourhoods, and who acted as trusted ‘brokers’. With the Departure Lounge, for example, the session with the most active engagement was in a small community café. The researcher had met with local staff and volunteers beforehand to explain the project, and the community workers were aware of local residents who had been bereaved, or who were living with life-limiting illness, and whom they felt might want to discuss experiences or concerns. They advertised the event beforehand *via* word of mouth and social media and encouraged people to attend. The community worker was present at the event, and introduced the researcher to residents, thereby encouraging interaction. This created an environment where people felt supported to talk about their own experiences, both to the researcher and to each other. Some people attended specifically seeking information.

Recruitment for focus groups was also carried out *via* organizations with existing relationships within communities. Three groups had higher attendance levels (six or seven participants, with three in the fourth group); in two of these most of the participants knew each other and were part of an established community group, and the third were all members of an established carers network, suggesting that familiarity with the group and/or trust in the hosting organization helped facilitate participation. Existing relationships were also key to recruitment to the Community Conversations.

#### Motivating factors for individual engagement

Often those who engaged with activities such as the Departure Lounge described a personal experience, such as caring for someone with a life-limiting illness or a bereavement, as the reason that they felt it was important to talk about dying. Some were currently dealing with related issues and saw the activity as an opportunity to seek information or discuss their situation. Another common motivator was an individual’s concern about their own future arrangements or care needs, or those of family members such as ageing parents.

Some individuals did not wish to engage with the Departure Lounge and chose to walk away once they became aware of the topic, stating that they either did not need or did not want to talk about death. Whilst this clearly varied depending on individual attitudes, there were also indications that local and cultural contexts could also play a part; for example, in the community café setting a recently established bereavement group had held their first meeting the day before the Departure Lounge visit, and this had prompted more conversations between regular visitors to the café about dying and bereavement. Conversely, when the Departure Lounge was at a community fun day attended by a range of local organizations, a representative from a local Chinese Community Association was interested in using the display within her own organization because she felt that the topic was considered taboo but wanted to encourage more open discussion.

#### Possible barriers to engagement

Whilst higher levels of engagement at some Departure Lounge events were clearly attributable to relational factors, underlying causes for lower levels of engagement at other venues can only be tentatively suggested from observations. At the Dying Matters event at a health centre, for instance, the small space available was dominated by the service providers running ‘stalls,’ making it difficult for members of the public to initiate private conversations and potentially overwhelming or intimidating to peruse the information.

Language was identified as a potential barrier at several events, with one community worker commenting that the use of the term ‘palliative care’ on a flyer excluded those unsure of its meaning. Several times during focus groups or Departure Lounge events people also commented that the word ‘hospice’ was generally perceived as ‘the place you go to die’, rather than as an organization offering information or support for those with life-limiting illnesses.

During the CBs focus group, one CB described how one of the Community Conversation participants had discussed with others at a coffee morning her positive experience of taking part in the Conversation. The CB was aware that a number of individuals attending the coffee morning had experienced bereavement and thought that this might encourage others to participate. However, the topic provoked negative reactions from several previous carers who expressed discomfort. The CBs concluded that the coffee morning was seen by some as a space to ‘escape’ from difficult experiences, and discussions about end of life were therefore perceived to be inappropriate:

R1 ‘just a coffee morning, yes, with a lot of people there who’d lost people and who’d been through end of life’.
R2: But they’re there to forget it.
R1: Exactly.
R2: The role of that particular group is leaving that behind, presumably, and she suddenly brought it in and that’s not the right space.
R1: No, no, they go there to have fun, to have a laugh, be friends.
[CB Focus Group]

This again suggest that the social norms within a group, and its context, contribute to willingness to address topics related to dying and bereavement.

### Limitations

The primary aim of our engagement programme was to explore the issues related to dying and bereavement that are important to people within rural, coastal and low-income communities to inform future research priorities. Therefore, these reflections are grounded in observations and collective ‘sense-making’, rather than seeking to evaluate different models of engagement.

Within the limited resources available, it was not possible to cover the large geography of the South-west Peninsula; hence, we purposefully sampled participants from specific areas of interest (i.e. coastal, rural inland, small towns, cities) with existing community networks. Time and resource limitations made it difficult to engage residents in the most isolated rural areas in which there is little existing community infrastructure. Future research should focus on these underserved areas and allow sufficient time to establish trusted contacts to facilitate engagement.

Whilst the Community Conversations were purposefully sited within the Torbay area, where CBs were able to make use of their longstanding connections with both individuals and community organizations to recruit participants, we acknowledge that the sample was more likely to include individuals with existing social connections within their neighbourhoods, and therefore perhaps not the most isolated.

The necessary extension of the timescale for the storytelling element of the project was due to both recruitment challenges, and the need to respect the ‘back and forth’ nature of the co-creation process and the pace that felt comfortable for participants. This also underlines the need to include generous timeframes when planning sensitive engagement work with individuals who may be experiencing challenging life circumstances such as bereavement.

### Key learning points and ideas for building community capacity

#### Valuing and supporting existing community assets

It was evident from discussions and observations that there are many community ‘assets’ that, whilst supporting people more generally, also support people through dying and bereavement. As well as the community cafes and well-being hub, this included churches and faith groups, and some less obvious sources of support, such as libraries. At one engagement event a library staff member commented that their ‘Better with a book’ sessions are often attended by people who have been bereaved, and they felt that this might be because they provide somewhere for people to come and connect with others without specifically seeking bereavement support. This reflects the finding from previous research that strong community support was an important element of end-of-life care for patients and caregivers in rural areas.^8^ This suggests that alongside specialist provision such as bereavement support groups and grief cafes, there is value in offering community groups and volunteers already embedded within neighbourhoods opportunities to develop their knowledge and skills to sensitively support people at the end of life and their carers. The experience of the CBs appears to confirm that this model of equipping those already working in the community with training and tools can help them feel secure in having conversations with people about dying and bereavement and to gain a better understanding of individuals’ needs.

#### Engaging underserved communities in research

As previously described, engagement levels were significantly higher, particularly in low-income areas, when the researcher had spent time talking with community workers and volunteers in a particular setting about the research before an activity took place, being guided by their knowledge in relation to suitable timings and venues and gaining their support for the recruitment and consent process. Conversations with experienced community workers also guided our data collection in other ways; for example, the tone of the focus groups was informal, with participants being able to arrive and leave during the session if they wished to and with a familiar face available to help people negotiate information sheets and consent forms. The usefulness of additional participant demographic information was weighed against the burden of paperwork or questioning, and so the data required was kept to a minimum. This appeared to be effective in terms of encouraging participation, as recruitment to focus groups as well as engagement with the Departure Lounge were higher in areas where this relationship building between the researcher and community workers had taken place beforehand.

#### Potential for partnership

In their report on ‘Accessing quality care in rural areas’, Marie Curie describe an innovative approach in rural Scotland in which community nurses work in partnership with stakeholders such as care homes, and statutory services, including transport planning, to deliver a more responsive service.^18^ Our research also supports this model of partnership working and highlights the benefits of including community or neighbourhood-based groups in such partnerships to reach underserved communities.

However, it is also important to acknowledge that not all community workers will want to engage with the topics of dying or bereavement, depending on their personal views and circumstances, and also that those who do can find it difficult, either because they have limited knowledge of where and how to signpost people for specialist support or because of the emotional toll. Palliative and bereavement care organizations could therefore play a key role in building capacity for end-of-life support within communities. This could include working with community workers and volunteers by, for example, offering training in talking about dying or bereavement, facilitating ongoing supervision or peer group support, or developing accessible routes to specialist information.

#### Co-creating sustainable and local solutions

Several groups generated ideas for initiatives that could build capacity within communities to address the issues or problems they had identified. In one group, for example, carers discussed the idea of a free advice clinic where people could get support to access disability benefits or obtain power of attorney. Another group discussed common misperceptions about the role of their local hospice, which they saw as a place that people go to die rather than a source of support and information at end of life. They suggested that it would be helpful if a hospice volunteer could make regular visits to ‘on the ground’ organizations such as their own, to bridge the perceived gap between the hospice and community. Whilst there may be some resource implications attached to these suggestions, and it may be difficult for small community groups to action them alone, these relatively small, local initiatives became conversations about feasible future projects within the partnership context. An Australian study of rural palliative care services found that in areas with exemplary service structures, these often relied on ‘*ad hoc* and informal relationships’, and noted that whilst ‘complex informal networks are a staple of rural healthcare’, there was a need for the integration of standardized guidelines for routine care.^19^ Whilst this is clearly an important factor for improving the quality of clinical care, it may also be helpful to consider the rich potential that these complex and informal networks may provide in terms of developing community capacity to support those at end of life. Indeed, a UK study of urban and rural differences in access to palliative care services recommends that end-of-life care policies and strategies consider differences in settlement types such as rurality^20^; the findings from both our engagement work and scoping review^16^ suggest that strategies to strengthen capacity for community support should also seek to develop tailored solutions that respond to local context.

In their exploration of the concept of community engagement in end-of-life care, Sallnow and Paul^12^ make a distinction between community engagement (in which professionals share knowledge or raise awareness) and community development, which leads to individual and community change. D’Eer *et al*’s^21^ systematic review of civic engagement initiatives in palliative care showed that efforts that were not initiated by, or did not heavily involve, local communities, were less sustainable. Our programme contained a spectrum of different types of engagement, and it is the initiative developed in partnership with embedded community workers that may have the most sustained impact. CBs expressed a desire to continue to build on their skills in addressing end of life and bereavement needs, as they recognized through the research the value that this could add to their work. As a result, the umbrella organization supporting them, TCDT, has formed a new partnership with a cancer charity to explore support needs in the community.

Leonard *et al*.^22^ stress the importance of death education in the context of community development that is ‘more about creating sustainable collaborations’ rather than a didactic approach concerned with informing or consulting. Their work on death literacy within communities describes it as a form of ‘practice wisdom’, where, for instance, carers are actively engaged in a critical learning process to develop the knowledge and skills demanded by their caring role, and this knowledge is then shared through their support networks. On a small scale, our PPI group demonstrated this process as experts by experience; they used their knowledge to inform and educate partnership members about community needs from their perspective. The funding for the partnership enabled the infrastructure to develop and support this connection between community partners, academics and clinical expertise. Whilst this has been invaluable in developing and delivering the aims of the partnership, it also raises questions as to how their engagement can be funded and supported as part of service development and research going forward.

Perhaps the clearest message from our engagement programme is the need for those wishing to promote a public health approach to palliative care to consider how best to create spaces for organizations with palliative care and bereavement expertise and existing community organizations with a broader community development remit, to develop a shared understanding of each other’s reach and roles, and to work in partnership to increase local capacity to support the dying and bereaved. Rather than seeking a universal model, layers of support that address local needs and gaps in service provision and build on existing assets can provide multiple access points and intentionally target populations such as those in rural and coastal areas who are traditionally underserved by current models of health care.

## Appendices

### Appendix 1: South-West Peninsula Palliative Care Research Partnership members

A full list of the organizations involved in the partnership is provided below:

Cornwall Hospice Care

Devon & Cornwall Police

Health Watch Devon, Plymouth & Torbay

Hospiscare

King’s College London

Marie Curie

North Devon Hospice

NIHR Clinical Research Network South-West Peninsula

NIHR Research Design Service South-West

Plymouth Octopus Project

Rowcroft Hospice

St Luke’s Hospice

St Margaret’s Hospice

Torbay Community Development Trust

University College London/St Christopher’s Hospice

University of Exeter

University of Plymouth.

### Appendix 2

**Table:** Table of themes and subthemes from the analysis of community engagement data.

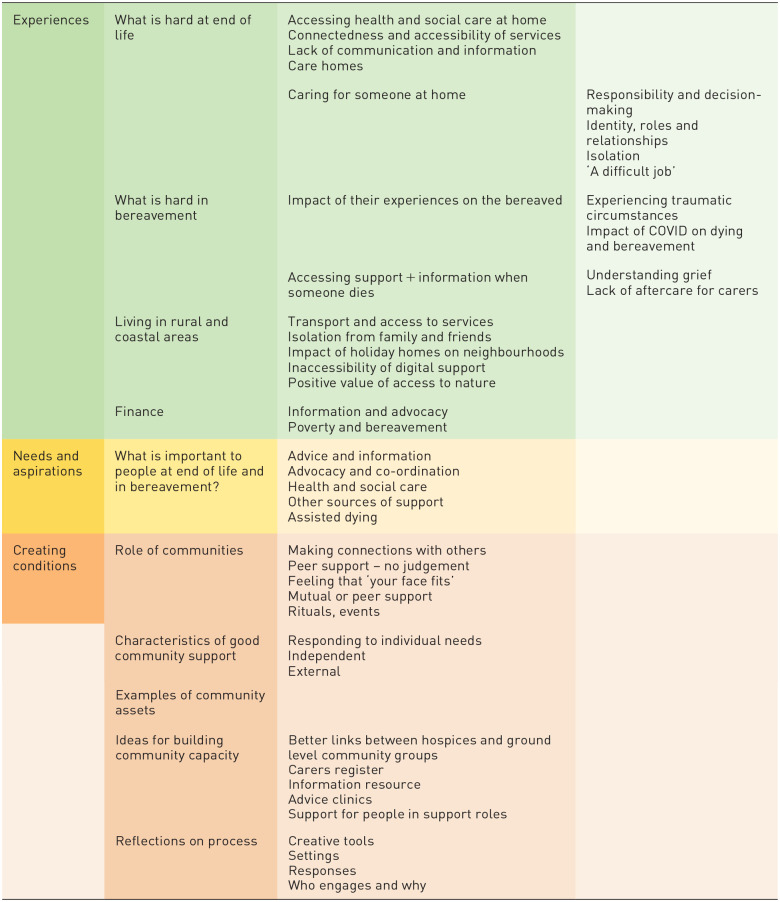

### Appendix 3 – Community Conversation toolkit

The photos below show examples of the suitcase activities, questions and recording sheet.

### Appendix 4

**Table:** Community Conversation participant demographics.

Age group		Gender		Ethnicity	
18–24	2	Male	6	White English/Welsh/Scottish/Northern Irish/British	26
25–34	1	Female	20		
35–44	0				
45–54	2				
55–64	13				
65–74	2				
75–84	3				
85–94	3				
Total	26	Total	26	Total	26

### Appendix 5

**Table:** Focus group participant demographics.

Age group		Gender		Ethnicity	
18–24	0	Male	5	White English/Welsh/Scottish/Northern Irish/British	17
25–34	3	Female	17	Gypsy or Irish Traveller	1
35–44	2			Multiple: White Peruvian	1
45–54	4			Other: Asian Filipino	1
55–64	9			White German	1
65–74	3			Other: prefer not to say	1
75–84	1				
85–94	0				
Total	22	Total	22	Total	22

### Appendix 6

**Focus groups – topic guide**

*Introduction*

Explain purpose of the discussion: We will talk about people’s experiences of accessing care, giving and receiving support, getting information and advice and what this is like where you live.

*Context*

Everyone here has different experiences – they may be living with an illness themselves, or caring for someone with an illness, or have been through a bereavement. Also, everyone will have had different experiences of care and support and may live in different areas with different resources available to them.

1. If you are happy to, could you briefly say a little bit about your own situation and what has brought you to the focus group (only if you would like to)

Prompt: For example, carer, bereaved and so on, whether accessed palliative care and so on.

2. As you know, our research is looking particularly at issues for people dealing with end-of-life experiences in rural, coastal and low-income areas in the south-west. Can we talk about any issues, problems or benefits you felt were related to living where you do?
3. In particular, were there any geographical issues that affected the care or support that you have received?

Prompt: For example, availability of transport, distance to services/organizations, availability of carers at home, rural isolation, neighbourhood community, distance to family and friends

*Accessing information, advice and support*

4. What has your experience been of getting the information or advice that you need?

Prompts: For example

- Understanding what will happen, knowing what to expect, processes and medical information
- Knowing what help is available and how to access it (palliative care, social care, practical help, advice on preparing for dying, advice on benefits, housing, finances, etc.)

*Support needs*

5. If you are living with illness, what kinds of information, advice or support have been helpful? Has there been anything missing that would have been helpful?
6. If you are a carer, what kinds of information, advice or support have been helpful? Has there been anything missing that would have been helpful?

Prompts: When caring, or when bereaved

7. Who would be best to provide this support?

Prompt: For example, does it need to be healthcare professionals, social care, community organizations, volunteers, neighbours and others who have been through something similar

*Community*

8. Would you reach out to your local community for support and if so what kind of support would be helpful?

Prompt: For example, friends, neighbours, local groups, faith groups, community centres, community centres, CBs and so on.

9. What do you think would make it easier for people to ask for help, or access the right support?

Prompt: What might make this a positive or negative experience for people?

10. Anything else you would like to say about what support is needed
- For individuals
- For communities

*AOB*

11. Any other thoughts/topics/ideas you would like to discuss or feedback today?

*Endin*

**Focus group for ‘Community Conversation’ facilitators – topic guide**

Introduction

Explain the purpose of the discussion: We will work through three key areas

- Overall learning from the conversations about palliative, and what themes are emerging
- Feedback on the ‘Community Conversations’ toolkit as a method/approach
- Your own experiences as facilitators – what it was like to talk to people about death and dying, and their experiences of support at end of life

*Discussion*

Introductions – ask everyone to give a brief summary of their involvement (how many interviews, ages, gender and situations)

Overall themes

1. From your experience, what would you say were the main themes or issues that came through from the interviews that you did?

Thinking about the individuals you spoke to. . .

2. What issues came out (barriers and facilitators) in terms of accessing care and support?

Prompts: Worries/fears? What are the biggest challenges people face? Positive aspects?

3. Do you think that issues/concerns/levels of support vary across social groups (age, culture and gender)? If so, in what way and why?

Prompts: Age, gender, socio-economic status and so on.

4. Similarly, do you think the issues and concerns vary in terms of neighbourhood or place?

Prompts: Low-income areas, more rural and coastal areas, isolation, and so on.

Thinking about the communities or neighbourhoods that you work in. . .

5. As someone who works as a CB, and has done these interviews, do you have any thoughts on how communities can be better supported or prepared to support individuals or families at end of life/bereavement? What resources do communities need to support dying well and grieving well?
6. What are the gaps?

Prompts: For example, training for community groups/leaders, more accessible information, advocacy, access to legal advice or healthcare advice

7. What resources are already in place that support dying well and grieving well? Are they being used, who uses them and why?
8. How do you think people feel about seeking support within their communities?

Prompts: Pros and cons? Feelings of stigma and dependence? Or valuing trusted relationships and combatting isolation

Thinking about the method – using the ‘Community Conversation’ toolkit

9. What did you like about using the toolkit? What worked well?

Prompts: What was it about this particular aspect/tool that worked well? Why?

10. Was there anything difficult or challenging about using the toolkit?

Prompts: What was it about this particular aspect/tool that was difficult? Why?

11. If we were designing this project again, what should we do differently?
12. Any other thoughts on using the toolkit?

Reflections on being the facilitator

13. What was it like for you, facilitating conversations about this sensitive topic?

Prompts: For example, recruitment, being afraid of upsetting someone, difficult conversations, dealing with own emotions and experiences

14. Do you have any thoughts on how people working in communities could be better supported to have conversations like these?

Prompts: For example, specific training, supervision and knowledge

15. What can we learn from this about how to better support communities in dealing with death and dying?

AOB

16. Any other thoughts/topics/ideas you would like to discuss or feedback today?

*Ending*

## Declarations

**Ethics approval and consent to participate:** The study was approved by the University of Exeter College of Humanities Ethics Committee (Ethical Clearance Reference Number: 493439) in January 2022. All Community Conversation, focus group and storytelling participants provided written informed consent prior to participating. Identifying information, such as names or specific locations, have been anonymized to ensure participant safety and privacy.

**Consent for publication:** All Community Conversation, focus group and storytelling participants provided written consent prior to participating in this study. The consent form explicitly outlined that participants’ anonymized verbatim quotes may be used in academic publications.

**Availability of data and materials:** All data requests should be submitted to the corresponding author for consideration. Access to anonymized data may be granted following review.

## References

[1] Office for National Statistics. Rural urban classification. Of lower layer super output areas in England and Wales, https://www.gov.uk/government/statistics/2011-rural-urban-classification-of-local-authority-and-other-higher-level-geographies-for-statistical-purposes. (2011, accessed 30 January 2023).

[2] Office for National Statistics. Subnational population projections for England: 2018-based, https://www.ons.gov.uk/peoplepopulationandcommunity/populationandmigration/populationprojections/bulletins/subnationalpopulationprojectionsforengland/2018based (2020, accessed 30 January 2023).

[3] Ministry of Housing CLG. English indices of deprivation 2019, https://www.gov.uk/government/statistics/english-indices-of-deprivation-2019 (2019, accessed 30 January 2023).

[4] Whitty C. Chief Medical Officer’s annual report 2021 health in coastal communities. London: Department of Health and Social Care, 2021.

[5] Hospice UK . Equality in hospice and end of life care: challenges and change. London, UK: Hospice, 2021.

[6] Tobin J Rogers A Winterburn I , et al. Hospice care access inequalities: a systematic review and narrative synthesis. BMJ Support Palliat Care 2022; 12: 142–151. 10.1136/bmjspcare-2020-002719 PMC9125370 33608254

[7] Rowley J Richards N Carduff E , et al. The impact of poverty and deprivation at the end of life: a critical review. Palliat Care Soc Pract 2021; 15: 26323524211033873. 34541536 10.1177/26323524211033873 PMC8442481

[8] Rainsford S MacLeod RD Glasgow NJ , et al. Rural end-of-life care from the experiences and perspectives of patients and family caregivers: a systematic literature review. Palliat Med 2017; 31: 895–912. 28106516 10.1177/0269216316685234

[9] Bonevski B Randell M Paul C , et al. Reaching the hard-to-reach: a systematic review of strategies for improving health and medical research with socially disadvantaged groups. BMC Med Res Methodol 2014; 14: 42. 24669751 10.1186/1471-2288-14-42 PMC3974746

[10] Sallnow L Richardson H Murray SA , et al. The impact of a new public health approach to end-of-life care: a systematic review. Palliat Med 2016; 30: 200–211. 26269324 10.1177/0269216315599869

[11] Grindrod A. Choice depends on options: a public health framework incorporating the social determinants of dying to create options at end of life. Prog Palliat Care 2020; 28: 94–100.

[12] Sallnow L Paul S. Understanding community engagement in end-of-life care: developing conceptual clarity. Crit Public Health 2015; 25: 231–238.

[13] Kellehear A. Compassionate communities: end-of-life care as everyone’s responsibility. QJM 2013; 106: 1071–1075. 24082152 10.1093/qjmed/hct200

[14] Hospice UK . Compassionate inverclyde, https://www.hospiceuk.org/innovation-hub/support-for-your-role/non-clinical-resources/community-volunteering/case-studies/compassionate-inverclyde (2023, accessed 01 September 2023).

[15] St Lukes Hospice. Plymouth, a compassionate city, https://www.stlukes-hospice.org.uk/plymouth-a-compassionate-city/ (2018, accessed 01 June 2023).

[16] Peeler A Doran A Winter-Dean L , et al. Public health palliative care interventions that enable communities to support people who are dying and their carers: a scoping review of studies that assess person-centered outcomes. Front Public Health 2023; 11: 1180571. 37564426 10.3389/fpubh.2023.1180571 PMC10410270

[17] Hansford L Wyatt K Creanor S , et al. Building a multi-sectoral palliative care research partnership in the South West Peninsula to understand community needs in rural, coastal and low-income areas. NIHR Public Health Research, In press. 10.3310/ATFA4287 38421270

[18] Marie Curie . Accessing quality care in rural areas: remote and rural Scotland partnership case study. https://www.mariecurie.org.uk/globalassets/media/documents/commissioning-our-services/partnership-case-studies/remote-and-rural-access-to-responsive-quality-care-in-rural-areas.pdf (accessed 30 March 2023).

[19] Disler R Pascoe A Hickson H , et al. Service level characteristics of rural palliative care for people with chronic disease. J Pain Symptom Manage 2023; 66: 301–309. 37343902 10.1016/j.jpainsymman.2023.06.003

[20] Chukwusa E Verne J Polato G , et al. Urban and rural differences in geographical accessibility to inpatient palliative and end-of-life (PEoLC) facilities and place of death: a national population-based study in England, UK. Int J Health Geogr 2019; 18: 8. 31060555 10.1186/s12942-019-0172-1 PMC6503436

[21] D’Eer L Quintiens B Van den Block L , et al. Civic engagement in serious illness, death, and loss: a systematic mixed-methods review. Palliat Med 2022; 36: 625–651. 35287517 10.1177/02692163221077850 PMC11910752

[22] Leonard R Noonan K Horsfall D , et al. Developing a death literacy index. Death Stud 2022; 46: 2110–2122. 34152939 10.1080/07481187.2021.1894268

